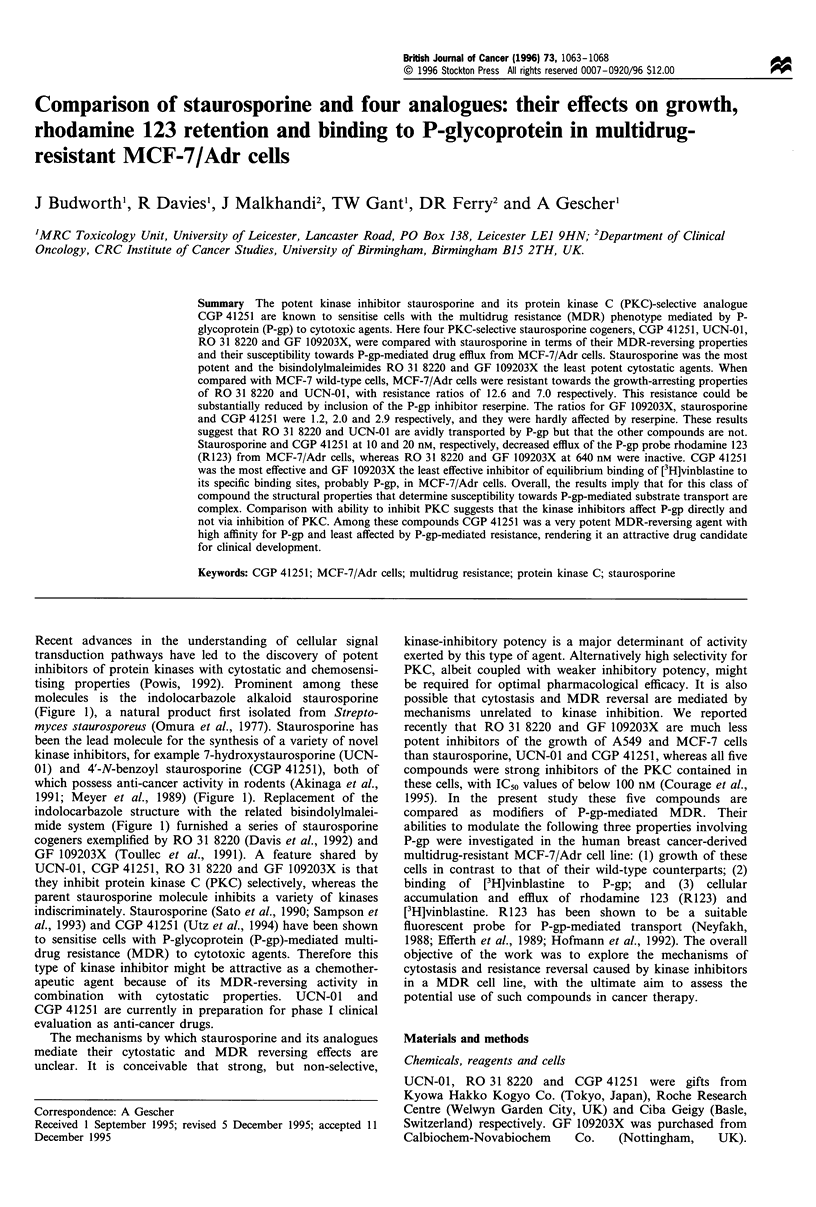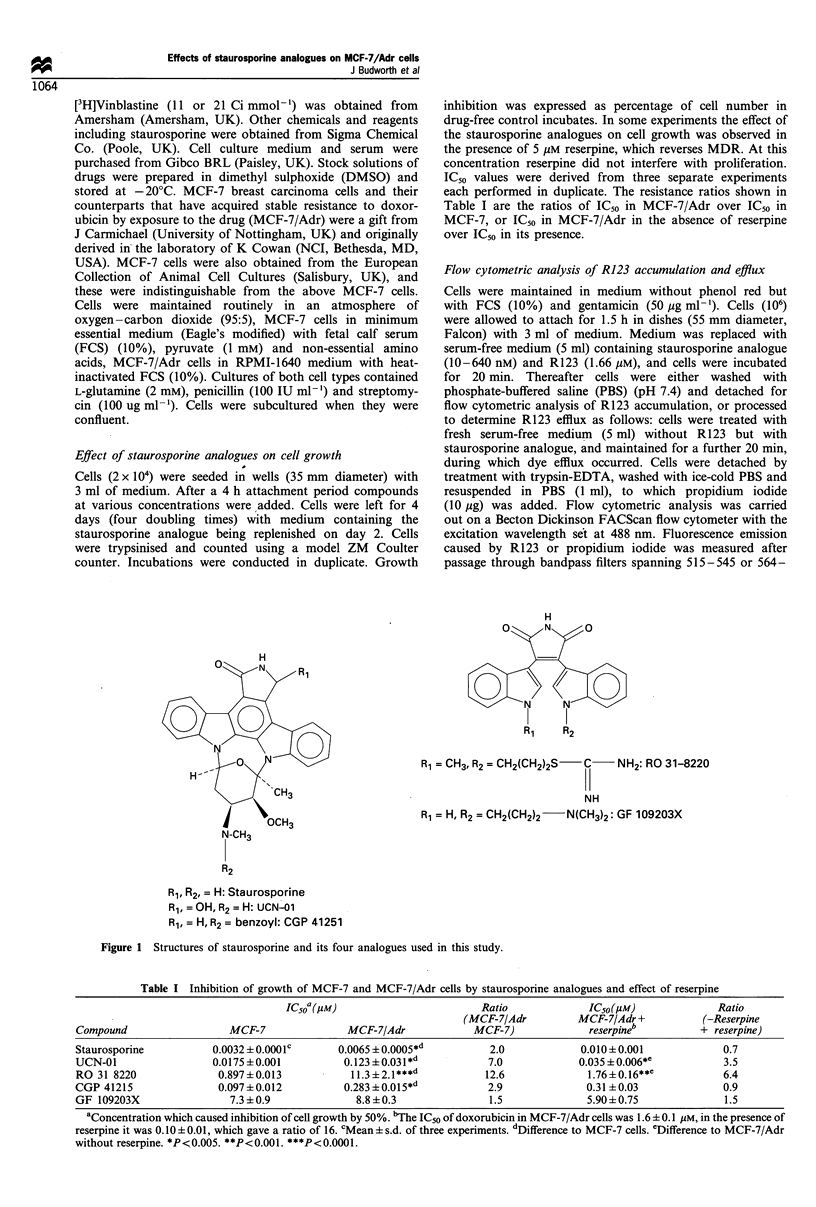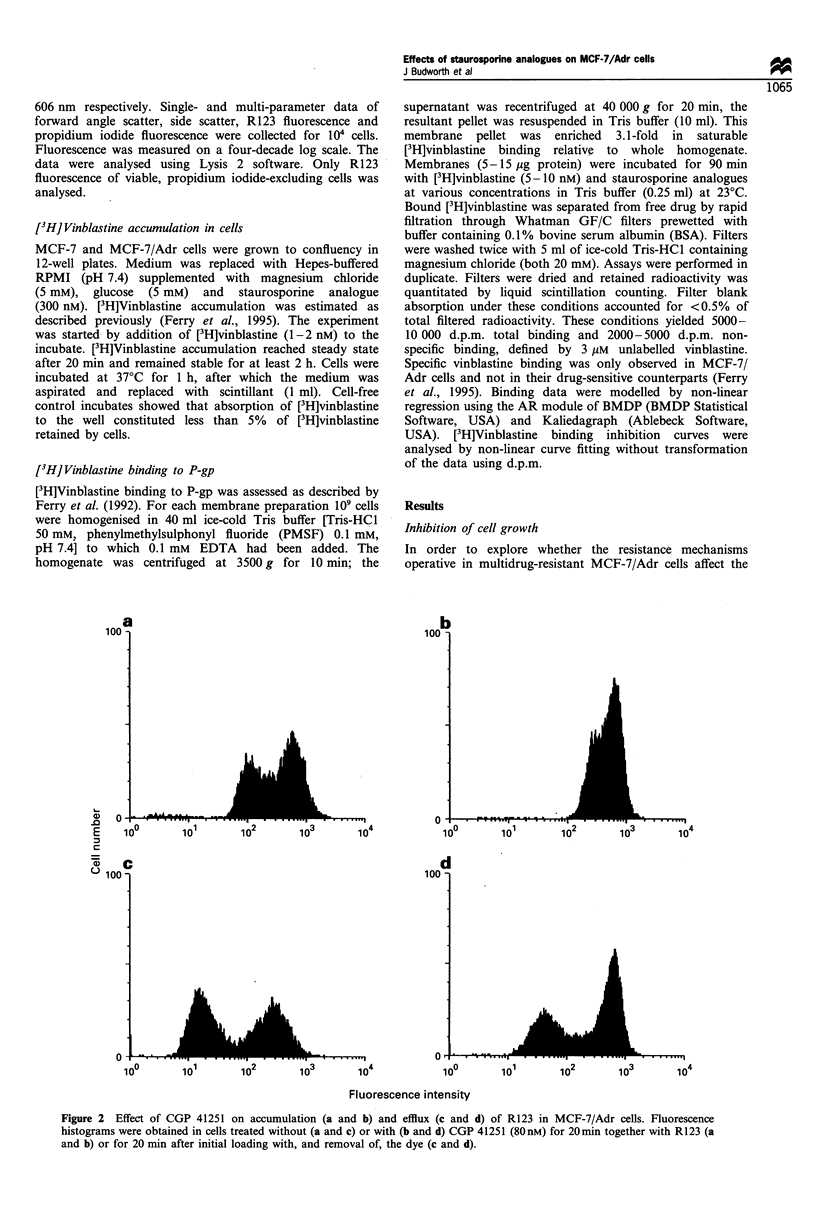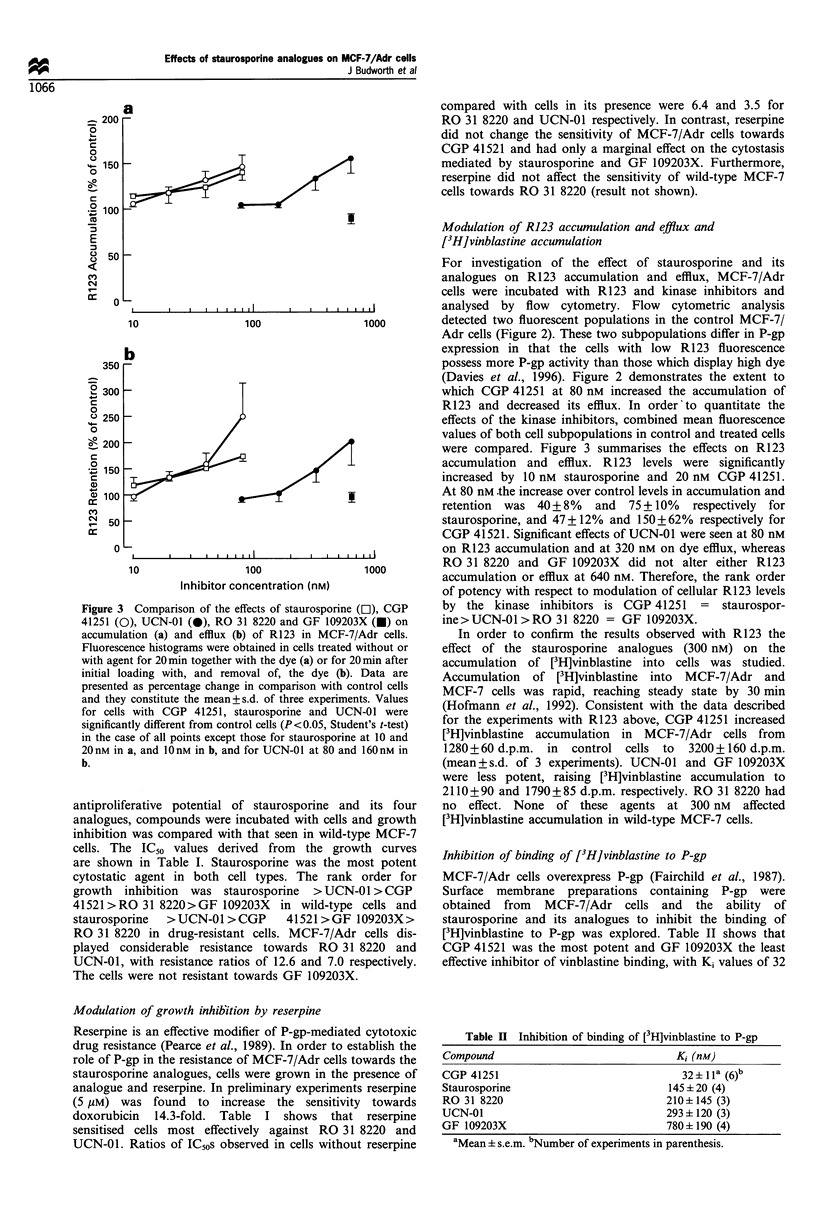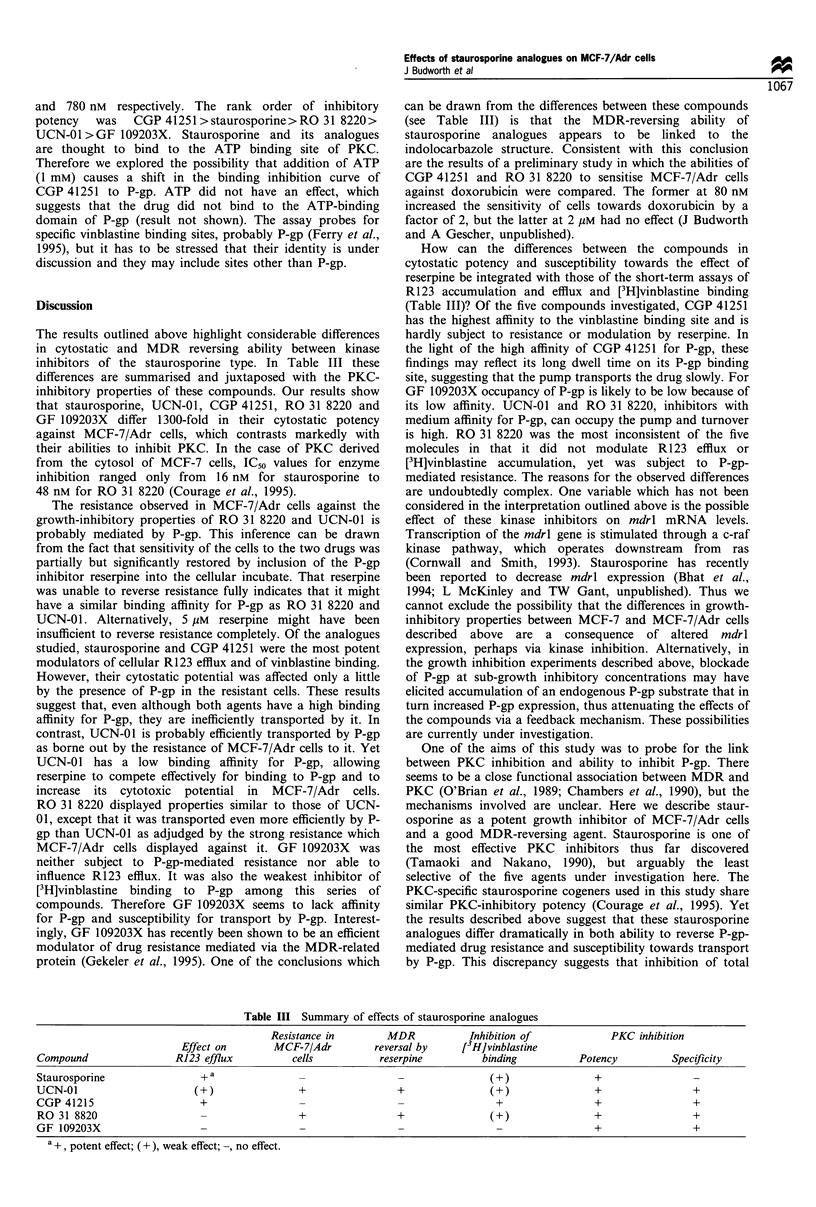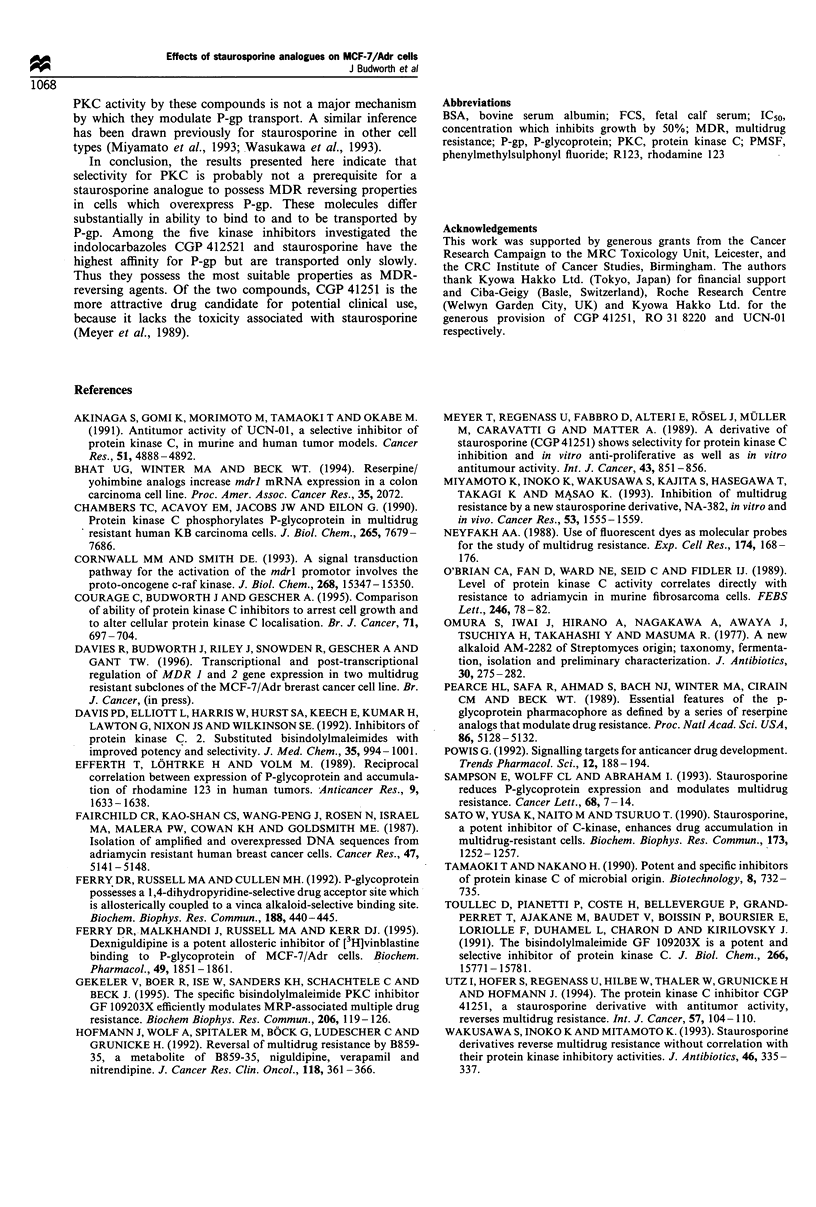# Comparison of staurosporine and four analogues: their effects on growth, rhodamine 123 retention and binding to P-glycoprotein in multidrug-resistant MCF-7/Adr cells.

**DOI:** 10.1038/bjc.1996.205

**Published:** 1996-05

**Authors:** J. Budworth, R. Davies, J. Malkhandi, T. W. Gant, D. R. Ferry, A. Gescher

**Affiliations:** MRC Toxicology Unit, University of Leicester, UK.

## Abstract

The potent kinase inhibitor staurosporine and its protein kinase C (PKC)-selective analogue CGP 41251 are known to sensitise cells with the multidrug resistance (MDR) phenotype mediated by P-glycoprotein (P-gp) to cytotoxic agents. Here four PKC-selective staurosporine cogeners, CGP 41251, UCN-01, RO 31 8220 and GF 109203X, were compared with staurosporine in terms of their MDR-reversing properties and their susceptibility towards P-gp-mediated drug efflux from MCF-7/Adr cells. Staurosporine was the most potent and the bisindolylmaleimides RO 31 8220 and GF 109203X the least potent cytostatic agents. When compared with MCF-7 wild-type cells, MCF-7/Adr cells were resistant towards the growth-arresting properties of RO 31 8220 and UCN-01, with resistance ratios of 12.6 and 7.0 respectively. This resistance could be substantially reduced by inclusion of the P-gp inhibitor reserpine. The ratios for GF 109203X, staurosporine and CGP 41251 were 1.2, 2.0 and 2.9 respectively, and they were hardly affected by reserpine. These results suggest that RO 31 8220 and UCN-01 are avidly transported by P-gp but that the other compounds are not. Staurosporine and CGP 41251 at 10 and 20 nM, respectively, decreased efflux of the P-gp probe rhodamine 123 (R123) from MCF-7/Adr cells, whereas RO 31 8220 and GF 109203X at 640 nM were inactive. CGP 41251 was the most effective and GF 109203X the least effective inhibitor of equilibrium binding of [3H]vinblastine to its specific binding sites, probably P-gp, in MCF-7/Adr cells. Overall, the results imply that for this class of compound the structural properties that determine susceptibility towards P-gp-mediated substrate transport are complex. Comparison with ability to inhibit PKC suggests that the kinase inhibitors affect P-gp directly and not via inhibition of PKC. Among these compounds CGP 41251 was a very potent MDR-reversing agent with high affinity for P-gp and least affected by P-gp-mediated resistance, rendering it an attractive drug candidate for clinical development.


British Journal of Cancer (1996) 73, 1063-1068
© 1996 Stockton Press All rights reserved 0007-0920/96 $12.00

### Comparison of staurosporine and four analogues: their effects on growth, rhodamine 123 retention and binding to P-glycoprotein in multidrug-resistant MCF-7/Adr cells

J Budworth[1], R Davies[1], J Malkhandi[2], TW Gant[1], DR Ferry[2] and A Gescher[1]

[1]MRC Toxicology Unit, University of Leicester, Lancaster Road, PO Box 138, Leicester LE1 9HN; [2]Department of Clinical Oncology, CRC Institute of Cancer Studies, University of Birmingham, Birmingham B15 2TH, UK.

**Summary** The potent kinase inhibitor staurosporine and its protein kinase C (PKC)-selective analogue CGP 41251 are known to sensitise cells with the multidrug resistance (MDR) phenotype mediated by P-glycoprotein (P-gp) to cytotoxic agents. Here four PKC-selective staurosporine cogeners, CGP 41251, UCN-01, RO 31 8220 and GF 109203X, were compared with staurosporine in terms of their MDR-reversing properties and their susceptibility towards P-gp-mediated drug efflux from MCF-7/Adr cells. Staurosporine was the most potent and the bisindolylmaleimides RO 31 8220 and GF 109203X the least potent cytostatic agents. When compared with MCF-7 wild-type cells, MCF-7/Adr cells were resistant towards the growth-arresting properties of RO 31 8220 and UCN-01, with resistance ratios of 12.6 and 7.0 respectively. This resistance could be substantially reduced by inclusion of the P-gp inhibitor reserpine. The ratios for GF 109203X, staurosporine and CGP 41251 were 1.2, 2.0 and 2.9 respectively, and they were hardly affected by reserpine. These results suggest that RO 31 8220 and UCN-01 are avidly transported by P-gp but that the other compounds are not. Staurosporine and CGP 41251 at 10 and 20 nM, respectively, decreased efflux of the P-gp probe rhodamine 123 (R123) from MCF-7/Adr cells, whereas RO 31 8220 and GF 109203X at 640 nM were inactive. CGP 41251 was the most effective and GF 109203X the least effective inhibitor of equilibrium binding of [$^3$H]vinblastine to its specific binding sites, probably P-gp, in MCF-7/Adr cells. Overall, the results imply that for this class of compound the structural properties that determine susceptibility towards P-gp-mediated substrate transport are complex. Comparison with ability to inhibit PKC suggests that the kinase inhibitors affect P-gp directly and not via inhibition of PKC. Among these compounds CGP 41251 was a very potent MDR-reversing agent with high affinity for P-gp and least affected by P-gp-mediated resistance, rendering it an attractive drug candidate for clinical development.

**Keywords:** CGP 41251; MCF-7/Adr cells; multidrug resistance; protein kinase C; staurosporine

Recent advances in the understanding of cellular signal transduction pathways have led to the discovery of potent inhibitors of protein kinases with cytostatic and chemosensitising properties (Powis, 1992). Prominent among these molecules is the indolocarbazole alkaloid staurosporine (Figure 1), a natural product first isolated from *Streptomyces staurosporeus* (Omura *et al.*, 1977). Staurosporine has been the lead molecule for the synthesis of a variety of novel kinase inhibitors, for example 7-hydroxystaurosporine (UCN-01) and 4'-*N*-benzoyl staurosporine (CGP 41251), both of which possess anti-cancer activity in rodents (Akinaga *et al.*, 1991; Meyer *et al.*, 1989) (Figure 1). Replacement of the indolocarbazole structure with the related bisindolylmaleimide system (Figure 1) furnished a series of staurosporine cogeners exemplified by RO 31 8220 (Davis *et al.*, 1992) and GF 109203X (Toullec *et al.*, 1991). A feature shared by UCN-01, CGP 41251, RO 31 8220 and GF 109203X is that they inhibit protein kinase C (PKC) selectively, whereas the parent staurosporine molecule inhibits a variety of kinases indiscriminately. Staurosporine (Sato *et al.*, 1990; Sampson *et al.*, 1993) and CGP 41251 (Utz *et al.*, 1994) have been shown to sensitise cells with P-glycoprotein (P-gp)-mediated multidrug resistance (MDR) to cytotoxic agents. Therefore this type of kinase inhibitor might be attractive as a chemotherapeutic agent because of its MDR-reversing activity in combination with cytostatic properties. UCN-01 and CGP 41251 are currently in preparation for phase I clinical evaluation as anti-cancer drugs.

The mechanisms by which staurosporine and its analogues mediate their cytostatic and MDR reversing effects are unclear. It is conceivable that strong, but non-selective, kinase-inhibitory potency is a major determinant of activity exerted by this type of agent. Alternatively high selectivity for PKC, albeit coupled with weaker inhibitory potency, might be required for optimal pharmacological efficacy. It is also possible that cytostasis and MDR reversal are mediated by mechanisms unrelated to kinase inhibition. We reported recently that RO 31 8220 and GF 109203X are much less potent inhibitors of the growth of A549 and MCF-7 cells than staurosporine, UCN-01 and CGP 41251, whereas all five compounds were strong inhibitors of the PKC contained in these cells, with $IC_{50}$ values of below 100 nM (Courage *et al.*, 1995). In the present study these five compounds are compared as modifiers of P-gp-mediated MDR. Their abilities to modulate the following three properties involving P-gp were investigated in the human breast cancer-derived multidrug-resistant MCF-7/Adr cell line: (1) growth of these cells in contrast to that of their wild-type counterparts; (2) binding of [$^3$H]vinblastine to P-gp; and (3) cellular accumulation and efflux of rhodamine 123 (R123) and [$^3$H]vinblastine. R123 has been shown to be a suitable fluorescent probe for P-gp-mediated transport (Neyfakh, 1988; Efferth *et al.*, 1989; Hofmann *et al.*, 1992). The overall objective of the work was to explore the mechanisms of cytostasis and resistance reversal caused by kinase inhibitors in a MDR cell line, with the ultimate aim to assess the potential use of such compounds in cancer therapy.

### Materials and methods

### *Chemicals, reagents and cells*

UCN-01, RO 31 8220 and CGP 41251 were gifts from Kyowa Hakko Kogyo Co. (Tokyo, Japan), Roche Research Centre (Welwyn Garden City, UK) and Ciba Geigy (Basle, Switzerland) respectively. GF 109203X was purchased from Calbiochem-Novabiochem Co. (Nottingham, UK).

Correspondence: A Gescher
Received 1 September 1995; revised 5 December 1995; accepted 11 December 1995

[$^3$H]Vinblastine (11 or 21 Ci mmol$^{-1}$) was obtained from Amersham (Amersham, UK). Other chemicals and reagents including staurosporine were obtained from Sigma Chemical Co. (Poole, UK). Cell culture medium and serum were purchased from Gibco BRL (Paisley, UK). Stock solutions of drugs were prepared in dimethyl sulphoxide (DMSO) and stored at −20°C. MCF-7 breast carcinoma cells and their counterparts that have acquired stable resistance to doxorubicin by exposure to the drug (MCF-7/Adr) were a gift from J Carmichael (University of Nottingham, UK) and originally derived in the laboratory of K Cowan (NCI, Bethesda, MD, USA). MCF-7 cells were also obtained from the European Collection of Animal Cell Cultures (Salisbury, UK), and these were indistinguishable from the above MCF-7 cells. Cells were maintained routinely in an atmosphere of oxygen–carbon dioxide (95:5), MCF-7 cells in minimum essential medium (Eagle's modified) with fetal calf serum (FCS) (10%), pyruvate (1 mM) and non-essential amino acids, MCF-7/Adr cells in RPMI-1640 medium with heat-inactivated FCS (10%). Cultures of both cell types contained L-glutamine (2 mM), penicillin (100 IU ml$^{-1}$) and streptomycin (100 ug ml$^{-1}$). Cells were subcultured when they were confluent.

*Effect of staurosporine analogues on cell growth*

Cells ($2 \times 10^4$) were seeded in wells (35 mm diameter) with 3 ml of medium. After a 4 h attachment period compounds at various concentrations were added. Cells were left for 4 days (four doubling times) with medium containing the staurosporine analogue being replenished on day 2. Cells were trypsinised and counted using a model ZM Coulter counter. Incubations were conducted in duplicate. Growth inhibition was expressed as percentage of cell number in drug-free control incubates. In some experiments the effect of the staurosporine analogues on cell growth was observed in the presence of 5 μM reserpine, which reverses MDR. At this concentration reserpine did not interfere with proliferation. $IC_{50}$ values were derived from three separate experiments each performed in duplicate. The resistance ratios shown in Table I are the ratios of $IC_{50}$ in MCF-7/Adr over $IC_{50}$ in MCF-7, or $IC_{50}$ in MCF-7/Adr in the absence of reserpine over $IC_{50}$ in its presence.

*Flow cytometric analysis of R123 accumulation and efflux*

Cells were maintained in medium without phenol red but with FCS (10%) and gentamicin (50 μg ml$^{-1}$). Cells ($10^6$) were allowed to attach for 1.5 h in dishes (55 mm diameter, Falcon) with 3 ml of medium. Medium was replaced with serum-free medium (5 ml) containing staurosporine analogue (10–640 nM) and R123 (1.66 μM), and cells were incubated for 20 min. Thereafter cells were either washed with phosphate-buffered saline (PBS) (pH 7.4) and detached for flow cytometric analysis of R123 accumulation, or processed to determine R123 efflux as follows: cells were treated with fresh serum-free medium (5 ml) without R123 but with staurosporine analogue, and maintained for a further 20 min, during which dye efflux occurred. Cells were detached by treatment with trypsin-EDTA, washed with ice-cold PBS and resuspended in PBS (1 ml), to which propidium iodide (10 μg) was added. Flow cytometric analysis was carried out on a Becton Dickinson FACScan flow cytometer with the excitation wavelength set at 488 nm. Fluorescence emission caused by R123 or propidium iodide was measured after passage through bandpass filters spanning 515–545 or 564–

**Figure 1** Structures of staurosporine and its four analogues used in this study.

**Table I** Inhibition of growth of MCF-7 and MCF-7/Adr cells by staurosporine analogues and effect of reserpine

| Compound | $IC_{50}$[a] (μM) MCF-7 | $IC_{50}$[a] (μM) MCF-7/Adr | Ratio (MCF-7/Adr / MCF-7) | $IC_{50}$ (μM) MCF-7/Adr + reserpine[b] | Ratio (−Reserpine / + reserpine) |
|---|---|---|---|---|---|
| Staurosporine | 0.0032 ± 0.0001[c] | 0.0065 ± 0.0005*[d] | 2.0 | 0.010 ± 0.001 | 0.7 |
| UCN-01 | 0.0175 ± 0.001 | 0.123 ± 0.031*[d] | 7.0 | 0.035 ± 0.006*[e] | 3.5 |
| RO 31 8220 | 0.897 ± 0.013 | 11.3 ± 2.1***[d] | 12.6 | 1.76 ± 0.16**[e] | 6.4 |
| CGP 41215 | 0.097 ± 0.012 | 0.283 ± 0.015*[d] | 2.9 | 0.31 ± 0.03 | 0.9 |
| GF 109203X | 7.3 ± 0.9 | 8.8 ± 0.3 | 1.5 | 5.90 ± 0.75 | 1.5 |

[a]Concentration which caused inhibition of cell growth by 50%. [b]The $IC_{50}$ of doxorubicin in MCF-7/Adr cells was 1.6 ± 0.1 μM, in the presence of reserpine it was 0.10 ± 0.01, which gave a ratio of 16. [c]Mean ± s.d. of three experiments. [d]Difference to MCF-7 cells. [e]Difference to MCF-7/Adr without reserpine. *$P$<0.005. **$P$<0.001. ***$P$<0.0001.

606 nm respectively. Single- and multi-parameter data of forward angle scatter, side scatter, R123 fluorescence and propidium iodide fluorescence were collected for $10^4$ cells. Fluorescence was measured on a four-decade log scale. The data were analysed using Lysis 2 software. Only R123 fluorescence of viable, propidium iodide-excluding cells was analysed.

*[³H]Vinblastine accumulation in cells*

MCF-7 and MCF-7/Adr cells were grown to confluency in 12-well plates. Medium was replaced with Hepes-buffered RPMI (pH 7.4) supplemented with magnesium chloride (5 mM), glucose (5 mM) and staurosporine analogue (300 nM). [$^3$H]Vinblastine accumulation was estimated as described previously (Ferry *et al.*, 1995). The experiment was started by addition of [$^3$H]vinblastine (1–2 nM) to the incubate. [$^3$H]Vinblastine accumulation reached steady state after 20 min and remained stable for at least 2 h. Cells were incubated at 37°C for 1 h, after which the medium was aspirated and replaced with scintillant (1 ml). Cell-free control incubates showed that absorption of [$^3$H]vinblastine to the well constituted less than 5% of [$^3$H]vinblastine retained by cells.

*[³H]Vinblastine binding to P-gp*

[$^3$H]Vinblastine binding to P-gp was assessed as described by Ferry *et al.* (1992). For each membrane preparation $10^9$ cells were homogenised in 40 ml ice-cold Tris buffer [Tris-HCl 50 mM, phenylmethylsulphonyl fluoride (PMSF) 0.1 mM, pH 7.4] to which 0.1 mM EDTA had been added. The homogenate was centrifuged at 3500 *g* for 10 min; the supernatant was recentrifuged at 40 000 *g* for 20 min, the resultant pellet was resuspended in Tris buffer (10 ml). This membrane pellet was enriched 3.1-fold in saturable [$^3$H]vinblastine binding relative to whole homogenate. Membranes (5–15 μg protein) were incubated for 90 min with [$^3$H]vinblastine (5–10 nM) and staurosporine analogues at various concentrations in Tris buffer (0.25 ml) at 23°C. Bound [$^3$H]vinblastine was separated from free drug by rapid filtration through Whatman GF/C filters prewetted with buffer containing 0.1% bovine serum albumin (BSA). Filters were washed twice with 5 ml of ice-cold Tris-HCl containing magnesium chloride (both 20 mM). Assays were performed in duplicate. Filters were dried and retained radioactivity was quantitated by liquid scintillation counting. Filter blank absorption under these conditions accounted for <0.5% of total filtered radioactivity. These conditions yielded 5000–10 000 d.p.m. total binding and 2000–5000 d.p.m. non-specific binding, defined by 3 μM unlabelled vinblastine. Specific vinblastine binding was only observed in MCF-7/Adr cells and not in their drug-sensitive counterparts (Ferry *et al.*, 1995). Binding data were modelled by non-linear regression using the AR module of BMDP (BMDP Statistical Software, USA) and Kaliedagraph (Ablebeck Software, USA). [$^3$H]Vinblastine binding inhibition curves were analysed by non-linear curve fitting without transformation of the data using d.p.m.

### Results

*Inhibition of cell growth*

In order to explore whether the resistance mechanisms operative in multidrug-resistant MCF-7/Adr cells affect the

**Figure 2** Effect of CGP 41251 on accumulation (**a** and **b**) and efflux (**c** and **d**) of R123 in MCF-7/Adr cells. Fluorescence histograms were obtained in cells treated without (**a** and **c**) or with (**b** and **d**) CGP 41251 (80 nM) for 20 min together with R123 (**a** and **b**) or for 20 min after initial loading with, and removal of, the dye (**c** and **d**).

**Figure 3** Comparison of the effects of staurosporine (□), CGP 41251 (○), UCN-01 (●), RO 31 8220 and GF 109203X (■) on accumulation (**a**) and efflux (**b**) of R123 in MCF-7/Adr cells. Fluorescence histograms were obtained in cells treated without or with agent for 20 min together with the dye (**a**) or for 20 min after initial loading with, and removal of, the dye (**b**). Data are presented as percentage change in comparison with control cells and they constitute the mean ± s.d. of three experiments. Values for cells with CGP 41251, staurosporine and UCN-01 were significantly different from control cells ($P < 0.05$, Student's $t$-test) in the case of all points except those for staurosporine at 10 and 20 nM in **a**, and 10 nM in **b**, and for UCN-01 at 80 and 160 nM in **b**.

antiproliferative potential of staurosporine and its four analogues, compounds were incubated with cells and growth inhibition was compared with that seen in wild-type MCF-7 cells. The $IC_{50}$ values derived from the growth curves are shown in Table I. Staurosporine was the most potent cytostatic agent in both cell types. The rank order for growth inhibition was staurosporine >UCN-01>CGP 41521>RO 31 8220>GF 109203X in wild-type cells and staurosporine >UCN-01>CGP 41521>GF 109203X> RO 31 8220 in drug-resistant cells. MCF-7/Adr cells displayed considerable resistance towards RO 31 8220 and UCN-01, with resistance ratios of 12.6 and 7.0 respectively. The cells were not resistant towards GF 109203X.

### *Modulation of growth inhibition by reserpine*

Reserpine is an effective modifier of P-gp-mediated cytotoxic drug resistance (Pearce *et al.*, 1989). In order to establish the role of P-gp in the resistance of MCF-7/Adr cells towards the staurosporine analogues, cells were grown in the presence of analogue and reserpine. In preliminary experiments reserpine (5 μM) was found to increase the sensitivity towards doxorubicin 14.3-fold. Table I shows that reserpine sensitised cells most effectively against RO 31 8220 and UCN-01. Ratios of $IC_{50}$s observed in cells without reserpine compared with cells in its presence were 6.4 and 3.5 for RO 31 8220 and UCN-01 respectively. In contrast, reserpine did not change the sensitivity of MCF-7/Adr cells towards CGP 41521 and had only a marginal effect on the cytostasis mediated by staurosporine and GF 109203X. Furthermore, reserpine did not affect the sensitivity of wild-type MCF-7 cells towards RO 31 8220 (result not shown).

### *Modulation of R123 accumulation and efflux and [$^3$H]vinblastine accumulation*

For investigation of the effect of staurosporine and its analogues on R123 accumulation and efflux, MCF-7/Adr cells were incubated with R123 and kinase inhibitors and analysed by flow cytometry. Flow cytometric analysis detected two fluorescent populations in the control MCF-7/Adr cells (Figure 2). These two subpopulations differ in P-gp expression in that the cells with low R123 fluorescence possess more P-gp activity than those which display high dye (Davies *et al.*, 1996). Figure 2 demonstrates the extent to which CGP 41251 at 80 nM increased the accumulation of R123 and decreased its efflux. In order to quantitate the effects of the kinase inhibitors, combined mean fluorescence values of both cell subpopulations in control and treated cells were compared. Figure 3 summarises the effects on R123 accumulation and efflux. R123 levels were significantly increased by 10 nM staurosporine and 20 nM CGP 41251. At 80 nM the increase over control levels in accumulation and retention was 40 ± 8% and 75 ± 10% respectively for staurosporine, and 47 ± 12% and 150 ± 62% respectively for CGP 41521. Significant effects of UCN-01 were seen at 80 nM on R123 accumulation and at 320 nM on dye efflux, whereas RO 31 8220 and GF 109203X did not alter either R123 accumulation or efflux at 640 nM. Therefore, the rank order of potency with respect to modulation of cellular R123 levels by the kinase inhibitors is CGP 41251 = staurosporine>UCN-01>RO 31 8220 = GF 109203X.

In order to confirm the results observed with R123 the effect of the staurosporine analogues (300 nM) on the accumulation of [$^3$H]vinblastine into cells was studied. Accumulation of [$^3$H]vinblastine into MCF-7/Adr and MCF-7 cells was rapid, reaching steady state by 30 min (Hofmann *et al.*, 1992). Consistent with the data described for the experiments with R123 above, CGP 41251 increased [$^3$H]vinblastine accumulation in MCF-7/Adr cells from 1280 ± 60 d.p.m. in control cells to 3200 ± 160 d.p.m. (mean ± s.d. of 3 experiments). UCN-01 and GF 109203X were less potent, raising [$^3$H]vinblastine accumulation to 2110 ± 90 and 1790 ± 85 d.p.m. respectively. RO 31 8220 had no effect. None of these agents at 300 nM affected [$^3$H]vinblastine accumulation in wild-type MCF-7 cells.

### *Inhibition of binding of [$^3$H]vinblastine to P-gp*

MCF-7/Adr cells overexpress P-gp (Fairchild *et al.*, 1987). Surface membrane preparations containing P-gp were obtained from MCF-7/Adr cells and the ability of staurosporine and its analogues to inhibit the binding of [$^3$H]vinblastine to P-gp was explored. Table II shows that CGP 41521 was the most potent and GF 109203X the least effective inhibitor of vinblastine binding, with $K_i$ values of 32

**Table II** Inhibition of binding of [$^3$H]vinblastine to P-gp

| *Compound* | $K_i$ *(nM)* |
|---|---|
| CGP 41251 | 32 ± 11[a] (6)[b] |
| Staurosporine | 145 ± 20 (4) |
| RO 31 8220 | 210 ± 145 (3) |
| UCN-01 | 293 ± 120 (3) |
| GF 109203X | 780 ± 190 (4) |

[a]Mean ± s.e.m. [b]Number of experiments in parenthesis.

and 780 nM respectively. The rank order of inhibitory potency was CGP 41251 > staurosporine > RO 31 8220 > UCN-01 > GF 109203X. Staurosporine and its analogues are thought to bind to the ATP binding site of PKC. Therefore we explored the possibility that addition of ATP (1 mM) causes a shift in the binding inhibition curve of CGP 41251 to P-gp. ATP did not have an effect, which suggests that the drug did not bind to the ATP-binding domain of P-gp (result not shown). The assay probes for specific vinblastine binding sites, probably P-gp (Ferry *et al.*, 1995), but it has to be stressed that their identity is under discussion and they may include sites other than P-gp.

### Discussion

The results outlined above highlight considerable differences in cytostatic and MDR reversing ability between kinase inhibitors of the staurosporine type. In Table III these differences are summarised and juxtaposed with the PKC-inhibitory properties of these compounds. Our results show that staurosporine, UCN-01, CGP 41251, RO 31 8220 and GF 109203X differ 1300-fold in their cytostatic potency against MCF-7/Adr cells, which contrasts markedly with their abilities to inhibit PKC. In the case of PKC derived from the cytosol of MCF-7 cells, $IC_{50}$ values for enzyme inhibition ranged only from 16 nM for staurosporine to 48 nM for RO 31 8220 (Courage *et al.*, 1995).

The resistance observed in MCF-7/Adr cells against the growth-inhibitory properties of RO 31 8220 and UCN-01 is probably mediated by P-gp. This inference can be drawn from the fact that sensitivity of the cells to the two drugs was partially but significantly restored by inclusion of the P-gp inhibitor reserpine into the cellular incubate. That reserpine was unable to reverse resistance fully indicates that it might have a similar binding affinity for P-gp as RO 31 8220 and UCN-01. Alternatively, 5 μM reserpine might have been insufficient to reverse resistance completely. Of the analogues studied, staurosporine and CGP 41251 were the most potent modulators of cellular R123 efflux and of vinblastine binding. However, their cytostatic potential was affected only a little by the presence of P-gp in the resistant cells. These results suggest that, even although both agents have a high binding affinity for P-gp, they are inefficiently transported by it. In contrast, UCN-01 is probably efficiently transported by P-gp as borne out by the resistance of MCF-7/Adr cells to it. Yet UCN-01 has a low binding affinity for P-gp, allowing reserpine to compete effectively for binding to P-gp and to increase its cytotoxic potential in MCF-7/Adr cells. RO 31 8220 displayed properties similar to those of UCN-01, except that it was transported even more efficiently by P-gp than UCN-01 as adjudged by the strong resistance which MCF-7/Adr cells displayed against it. GF 109203X was neither subject to P-gp-mediated resistance nor able to influence R123 efflux. It was also the weakest inhibitor of [$^3$H]vinblastine binding to P-gp among this series of compounds. Therefore GF 109203X seems to lack affinity for P-gp and susceptibility for transport by P-gp. Interestingly, GF 109203X has recently been shown to be an efficient modulator of drug resistance mediated via the MDR-related protein (Gekeler *et al.*, 1995). One of the conclusions which can be drawn from the differences between these compounds (see Table III) is that the MDR-reversing ability of staurosporine analogues appears to be linked to the indolocarbazole structure. Consistent with this conclusion are the results of a preliminary study in which the abilities of CGP 41251 and RO 31 8220 to sensitise MCF-7/Adr cells against doxorubicin were compared. The former at 80 nM increased the sensitivity of cells towards doxorubicin by a factor of 2, but the latter at 2 μM had no effect (J Budworth and A Gescher, unpublished).

How can the differences between the compounds in cytostatic potency and susceptibility towards the effect of reserpine be integrated with those of the short-term assays of R123 accumulation and efflux and [$^3$H]vinblastine binding (Table III)? Of the five compounds investigated, CGP 41251 has the highest affinity to the vinblastine binding site and is hardly subject to resistance or modulation by reserpine. In the light of the high affinity of CGP 41251 for P-gp, these findings may reflect its long dwell time on its P-gp binding site, suggesting that the pump transports the drug slowly. For GF 109203X occupancy of P-gp is likely to be low because of its low affinity. UCN-01 and RO 31 8220, inhibitors with medium affinity for P-gp, can occupy the pump and turnover is high. RO 31 8220 was the most inconsistent of the five molecules in that it did not modulate R123 efflux or [$^3$H]vinblastine accumulation, yet was subject to P-gp-mediated resistance. The reasons for the observed differences are undoubtedly complex. One variable which has not been considered in the interpretation outlined above is the possible effect of these kinase inhibitors on *mdr*1 mRNA levels. Transcription of the *mdr*1 gene is stimulated through a c-raf kinase pathway, which operates downstream from ras (Cornwall and Smith, 1993). Staurosporine has recently been reported to decrease *mdr*1 expression (Bhat *et al.*, 1994; L McKinley and TW Gant, unpublished). Thus we cannot exclude the possibility that the differences in growth-inhibitory properties between MCF-7 and MCF-7/Adr cells described above are a consequence of altered *mdr*1 expression, perhaps via kinase inhibition. Alternatively, in the growth inhibition experiments described above, blockade of P-gp at sub-growth inhibitory concentrations may have elicited accumulation of an endogenous P-gp substrate that in turn increased P-gp expression, thus attenuating the effects of the compounds via a feedback mechanism. These possibilities are currently under investigation.

One of the aims of this study was to probe for the link between PKC inhibition and ability to inhibit P-gp. There seems to be a close functional association between MDR and PKC (O'Brian *et al.*, 1989; Chambers *et al.*, 1990), but the mechanisms involved are unclear. Here we describe staurosporine as a potent growth inhibitor of MCF-7/Adr cells and a good MDR-reversing agent. Staurosporine is one of the most effective PKC inhibitors thus far discovered (Tamaoki and Nakano, 1990), but arguably the least selective of the five agents under investigation here. The PKC-specific staurosporine cogeners used in this study share similar PKC-inhibitory potency (Courage *et al.*, 1995). Yet the results described above suggest that these staurosporine analogues differ dramatically in both ability to reverse P-gp-mediated drug resistance and susceptibility towards transport by P-gp. This discrepancy suggests that inhibition of total

**Table III** Summary of effects of staurosporine analogues

| Compound | *Effect on R123 efflux* | *Resistance in MCF-7/Adr cells* | *MDR reversal by reserpine* | *Inhibition of [$^3$H]vinblastine binding* | *PKC inhibition* | |
|---|---|---|---|---|---|---|
| | | | | | *Potency* | *Specificity* |
| Staurosporine | +[a] | − | − | (+) | + | − |
| UCN-01 | (+) | + | + | (+) | + | + |
| CGP 41215 | + | − | − | + | + | + |
| RO 31 8820 | − | + | + | (+) | + | + |
| GF 109203X | − | − | − | − | + | + |

[a] +, potent effect; (+), weak effect; −, no effect.

PKC activity by these compounds is not a major mechanism by which they modulate P-gp transport. A similar inference has been drawn previously for staurosporine in other cell types (Miyamato et al., 1993; Wasukawa et al., 1993).

In conclusion, the results presented here indicate that selectivity for PKC is probably not a prerequisite for a staurosporine analogue to possess MDR reversing properties in cells which overexpress P-gp. These molecules differ substantially in ability to bind to and to be transported by P-gp. Among the five kinase inhibitors investigated the indolocarbazoles CGP 412521 and staurosporine have the highest affinity for P-gp but are transported only slowly. Thus they possess the most suitable properties as MDR-reversing agents. Of the two compounds, CGP 41251 is the more attractive drug candidate for potential clinical use, because it lacks the toxicity associated with staurosporine (Meyer et al., 1989).

**Abbreviations**

BSA, bovine serum albumin; FCS, fetal calf serum; $IC_{50}$, concentration which inhibits growth by 50%; MDR, multidrug resistance; P-gp, P-glycoprotein; PKC, protein kinase C; PMSF, phenylmethylsulphonyl fluoride; R123, rhodamine 123

**Acknowledgements**

This work was supported by generous grants from the Cancer Research Campaign to the MRC Toxicology Unit, Leicester, and the CRC Institute of Cancer Studies, Birmingham. The authors thank Kyowa Hakko Ltd. (Tokyo, Japan) for financial support and Ciba-Geigy (Basle, Switzerland), Roche Research Centre (Welwyn Garden City, UK) and Kyowa Hakko Ltd. for the generous provision of CGP 41251, RO 31 8220 and UCN-01 respectively.

### References

AKINAGA S, GOMI K, MORIMOTO M, TAMAOKI T AND OKABE M. (1991). Antitumor activity of UCN-01, a selective inhibitor of protein kinase C, in murine and human tumor models. *Cancer Res.*, **51**, 4888–4892.

BHAT UG, WINTER MA AND BECK WT. (1994). Reserpine/yohimbine analogs increase *mdr1* mRNA expression in a colon carcinoma cell line. *Proc. Amer. Assoc. Cancer Res.*, **35**, 2072.

CHAMBERS TC, ACAVOY EM, JACOBS JW AND EILON G. (1990). Protein kinase C phosphorylates P-glycoprotein in multidrug resistant human KB carcinoma cells. *J. Biol. Chem.*, **265**, 7679–7686.

CORNWALL MM AND SMITH DE. (1993). A signal transduction pathway for the activation of the *mdr*1 promotor involves the proto-oncogene c-raf kinase. *J. Biol. Chem.*, **268**, 15347–15350.

COURAGE C, BUDWORTH J AND GESCHER A. (1995). Comparison of ability of protein kinase C inhibitors to arrest cell growth and to alter cellular protein kinase C localisation. *Br. J. Cancer*, **71**, 697–704.

DAVIES R, BUDWORTH J, RILEY J, SNOWDEN R, GESCHER A AND GANT TW. (1996). Transcriptional and post-transcriptional regulation of *MDR 1* and *2* gene expression in two multidrug resistant subclones of the MCF-7/Adr brerast cancer cell line. *Br. J. Cancer*, (in press).

DAVIS PD, ELLIOTT L, HARRIS W, HURST SA, KEECH E, KUMAR H, LAWTON G, NIXON JS AND WILKINSON SE. (1992). Inhibitors of protein kinase C. 2. Substituted bisindolylmaleimides with improved potency and selectivity. *J. Med. Chem.*, **35**, 994–1001.

EFFERTH T, LÖHTRKE H AND VOLM M. (1989). Reciprocal correlation between expression of P-glycoprotein and accumulation of rhodamine 123 in human tumors. *Anticancer Res.*, **9**, 1633–1638.

FAIRCHILD CR, KAO-SHAN CS, WANG-PENG J, ROSEN N, ISRAEL MA, MALERA PW, COWAN KH AND GOLDSMITH ME. (1987). Isolation of amplified and overexpressed DNA sequences from adriamycin resistant human breast cancer cells. *Cancer Res.*, **47**, 5141–5148.

FERRY DR, RUSSELL MA AND CULLEN MH. (1992). P-glycoprotein possesses a 1,4-dihydropyridine-selective drug acceptor site which is allosterically coupled to a vinca alkaloid-selective binding site. *Biochem. Biophys. Res. Commun.*, **188**, 440–445.

FERRY DR, MALKHANDI J, RUSSELL MA AND KERR DJ. (1995). Dexniguldipine is a potent allosteric inhibitor of [$^3$H]vinblastine binding to P-glycoprotein of MCF-7/Adr cells. *Biochem. Pharmacol.*, **49**, 1851–1861.

GEKELER V, BOER R, ISE W, SANDERS KH, SCHACHTELE C AND BECK J. (1995). The specific bisindolylmaleimide PKC inhibitor GF 109203X efficiently modulates MRP-associated multiple drug resistance. *Biochem Biophys. Res. Commun.*, **206**, 119–126.

HOFMANN J, WOLF A, SPITALER M, BÖCK G, LUDESCHER C AND GRUNICKE H. (1992). Reversal of multidrug resistance by B859-35, a metabolite of B859-35, niguldipine, verapamil and nitrendipine. *J. Cancer Res. Clin. Oncol.*, **118**, 361–366.

MEYER T, REGENASS U, FABBRO D, ALTERI E, RÖSEL J, MÜLLER M, CARAVATTI G AND MATTER A. (1989). A derivative of staurosporine (CGP 41251) shows selectivity for protein kinase C inhibition and *in vitro* anti-proliferative as well as *in vitro* antitumour activity. *Int. J. Cancer*, **43**, 851–856.

MIYAMOTO K, INOKO K, WAKUSAWA S, KAJITA S, HASEGAWA T, TAKAGI K AND MASAO K. (1993). Inhibition of multidrug resistance by a new staurosporine derivative, NA-382, *in vitro* and *in vivo*. *Cancer Res.*, **53**, 1555–1559.

NEYFAKH AA. (1988). Use of fluorescent dyes as molecular probes for the study of multidrug resistance. *Exp. Cell Res.*, **174**, 168–176.

O'BRIAN CA, FAN D, WARD NE, SEID C AND FIDLER IJ. (1989). Level of protein kinase C activity correlates directly with resistance to adriamycin in murine fibrosarcoma cells. *FEBS Lett.*, **246**, 78–82.

OMURA S, IWAI J, HIRANO A, NAGAKAWA A, AWAYA J, TSUCHIYA H, TAKAHASHI Y AND MASUMA R. (1977). A new alkaloid AM-2282 of Streptomyces origin; taxonomy, fermentation, isolation and preliminary characterization. *J. Antibiotics*, **30**, 275–282.

PEARCE HL, SAFA R, AHMAD S, BACH NJ, WINTER MA, CIRAIN CM AND BECK WT. (1989). Essential features of the p-glycoprotein pharmacophore as defined by a series of reserpine analogs that modulate drug resistance. *Proc. Natl Acad. Sci. USA*, **86**, 5128–5132.

POWIS G. (1992). Signalling targets for anticancer drug development. *Trends Pharmacol. Sci.*, **12**, 188–194.

SAMPSON E, WOLFF CL AND ABRAHAM I. (1993). Staurosporine reduces P-glycoprotein expression and modulates multidrug resistance. *Cancer Lett.*, **68**, 7–14.

SATO W, YUSA K, NAITO M AND TSURUO T. (1990). Staurosporine, a potent inhibitor of C-kinase, enhances drug accumulation in multidrug-resistant cells. *Biochem. Biophys. Res. Commun.*, **173**, 1252–1257.

TAMAOKI T AND NAKANO H. (1990). Potent and specific inhibitors of protein kinase C of microbial origin. *Biotechnology*, **8**, 732–735.

TOULLEC D, PIANETTI P, COSTE H, BELLEVERGUE P, GRAND-PERRET T, AJAKANE M, BAUDET V, BOISSIN P, BOURSIER E, LORIOLLE F, DUHAMEL L, CHARON D AND KIRILOVSKY J. (1991). The bisindolylmaleimide GF 109203X is a potent and selective inhibitor of protein kinase C. *J. Biol. Chem.*, **266**, 15771–15781.

UTZ I, HOFER S, REGENASS U, HILBE W, THALER W, GRUNICKE H AND HOFMANN J. (1994). The protein kinase C inhibitor CGP 41251, a staurosporine derivative with antitumor activity, reverses multidrug resistance. *Int. J. Cancer*, **57**, 104–110.

WAKUSAWA S, INOKO K AND MITAMOTO K. (1993). Staurosporine derivatives reverse multidrug resistance without correlation with their protein kinase inhibitory activities. *J. Antibiotics*, **46**, 335–337.